# Using a liver-on-chip model to evaluate the effects of vancomycin and daptomycin on host immunometabolism and clearance of intracellular reservoirs of *Staphylococcus aureus* during bacteremia

**DOI:** 10.1128/aac.00254-26

**Published:** 2026-07-23

**Authors:** Brent Beadell, Sujung Yi, Makayla Roberts, Kyu Shik Mun, Annie Wong-Beringer

**Affiliations:** 1 Alfred E. Mann School of Pharmacy and Pharmaceutical Sciences, University of Southern California 5116, Los Angeles, California, USA; 2 Division of Pulmonary and Critical Care Medicine, Department of Medicine, Cedars-Sinai Medical Center 22494, Los Angeles, California, USA; 3 Board of Governors Regenerative Medicine Institute, Department of Pediatrics, Cedars-Sinai Medical Center, Los Angeles, California, USA; 4 Department of Pharmacy, Huntington Health 24296, Pasadena, California, USA; The Peter Doherty Institute for Infection and Immunity, Melbourne, Victoria, Australia

**Keywords:** daptomycin, vancomycin, Kupffer cell, liver, organ-on-chip, bacteremia, *S. aureus*

## Abstract

*Staphylococcus aureus* bacteremia (SAB) causes significant morbidity and mortality, despite treatment with guideline-recommended antibiotics. A critical contributor to SAB treatment failure is the intracellular persistence of SA within liver sinusoidal macrophages (Kupffer cells [KCs]). Antibiotics have been shown to modulate immune cell function, which is governed by cellular metabolism; yet, the effect of antibiotics on host immunometabolic response relative to bacterial clearance is understudied. We developed a liver-on-chip (LoC) model of SAB to recapitulate the liver sinusoid during systemic infection using exclusively liver-derived cells and the GFP USA300 LAC SA strain and compared the effects of vancomycin and daptomycin on host immunometabolism by fluorescence lifetime imaging microscopy and related to intracellular antimicrobial efficacy. Utilizing both murine and human-based LoC infection models, we found that DAP was superior to VAN treatment in clearing intracellular SA from the liver microenvironment, which corresponded to differential host immunometabolic responses. DAP showed low NAD(P)H fluorescence lifetime and low mitochondrial ROS, suggestive of a redox-buffered state that balanced pathogen containment with hepatic metabolic homeostasis, while VAN treatment biased towards greater utilization of OXPHOS. Our findings using a novel *in vitro* microphysiological system mimicking the liver tissue microenvironment offer insights into antibiotic-mediated bacterial clearance that accounts for host immunometabolism beyond direct antimicrobial activity.

## INTRODUCTION

Mortality rates from *S. aureus* bacteremia (SAB) remains high despite treatment with guideline-recommended antibiotics, which underscores the urgent need to improve our understanding of the disease pathogenesis to make meaningful improvements to therapeutic approaches. Importantly, immune cell function has been shown to be governed by cellular metabolism (1). While antibiotics have been shown to differentially modulate immunometabolism (2, 3), the link between antibiotic-mediated immunometabolic responses and intracellular clearance of SA from the liver reservoir has not been investigated. Traditional methods for studying SAB face several limitations that ultimately impact the translatability of their findings. Moreover, an immune cell’s metabolic trajectory (glycolytic or oxidative states) directly impacts their capacity for phagocytosis, microbicidal activity, and cytokine production, thereby governing bacterial clearance outcomes (4–6). *In vitro* models do not recapitulate the full biological complexity of SAB. Most of the published *in vitro* findings are based on studies performed in cell culture models with macrophages and/or neutrophils, which do not account for the contributions of crosstalk between other cells at the tissue level to infection outcomes and in static conditions without the mechanobiological forces from flow conditions that are known to shape both host and bacterial cellular metabolism and function (7). Murine models of SAB address many limitations of *in vitro* research, namely, by retaining biological complexity, but they also have well-described shortcomings (8–10). A major drawback is that mice are not a natural host for SA; as such, current experimental models require a high bacterial inoculum to establish bloodstream infection (11). In contrast, patients with SAB have a relatively low inoculum in the circulation with a range of 10^1^–10^3^ cfu/mL (12, 13). With the recent discovery of the pathogenic reservoir within KCs in the liver sinusoid during SAB and its contribution to persistence, modeling the whole liver sinusoid under flow conditions would provide a more physiological microenvironment from which to evaluate therapeutics targeting this intracellular reservoir of SA during bacteremia (14).

Previous studies and reviews have demonstrated that complex co-culture models that more closely recapitulate liver physiology can offer novel insights into disease pathology, including in infectious disease contexts (15–18). Collectively, there is significant evidence supporting the rationale for utilizing complex multicellular *in vitro* models of the liver sinusoid to advance our understanding of SA pathogenesis within the liver. The recent emergence of organ-on-a-chip (OoC) platforms as an attractive alternative to animals for human disease modeling presents a unique opportunity to apply these advanced *in vitro* models to deepen our understanding of the pathogenesis of SAB. These OoC systems can better emulate human physiological conditions at the organ of interest and enable increased resolution for studying cellular dynamics within them (19–21). Here, we sought to develop a LoC model comprised of liver sinusoidal endothelial cells (LSECs), hepatic stellate cells (HSCs), and KCs as the major non-parenchymal resident cells for the study of SAB that focuses on the pathogenic reservoir in the liver sinusoid, adapted from previously established LoC models (21, 22).

We previously described the differential physicochemical contributions of guideline-recommended antibiotics, vancomycin (VAN) and daptomycin (DAP) in targeting intracellular SA within KCs, as well as their impact on KC metabolism during infection *in vitro* (2). Here, we expanded our investigation to examine the effects of antibiotic treatment on host immunometabolism in relation to clearance of intracellular SA in a more physiologically relevant environment using the OoC platform. Immunometabolic response was evaluated by NAD(*P*)H fluorescence lifetime imaging microscopy (FLIM) and mitochondrial ROS production to assess the metabolic impact of vancomycin (VAN), the first-line antibiotic for the empiric treatment of MRSA bacteremia, and an alternative, daptomycin (DAP), while the use of a GFP-producing SA strain allows for the spatiotemporal tracking of the bacterium during the course of the infection and assessment of treatment efficacy at the organ level. We first utilized murine cells to establish the infection model and for initial evaluation, followed by confirmation of antibiotic intracellular efficacy in a human-based chip model.

## MATERIALS AND METHODS

### Bacterial preparation

Glycerol stocks of GFP USA300 LAC were grown on tryptic soy agar at 37°C overnight, followed by subculture of three representative colony-forming units (CFU) in tryptic soy broth for 16–20 h at 37°C, 250 rpm prior to use in co-infection assays.

### Cell culture conditions

For murine liver-on-chip experiments, an immortalized Kupffer cell (KC) line was utilized in combination with an immortalized sinusoidal endothelial cell (LSEC) line and a HSC line (Applied Biological Materials, Canada), which were both established from C57BL/6 mice (23). All cell types were maintained separately in DMEM (Thermo Fisher, MA, USA) +10% FBS (Genesee Scientific, CA, USA) +10 µg/mL bovine insulin (Sigma-Aldrich, MO, USA) +250 µM monothioglycerol (Sigma-Aldrich, MO, USA) prior to seeding in liver chips for experimentation. For human liver-on-chip experiments, primary LSECs (Cell Systems, WA, USA) and HSCs (Zen-Bio, NC, USA) were purchased and cultured for two days prior to liver chip seeding. LSECs were cultured in Complete Classic Medium With Serum and CultureBoost (Cell Systems, WA, USA) supplemented with Bac-off (Cell Systems, WA, USA). HSCs were cultured in Human Stellate Cell Growth Medium (Zen-Bio, NC, USA). Primary human KCs (Zen-Bio, NC, USA) were purchased and thawed immediately prior to liver chip seeding.

### Antibiotic preparation

Vancomycin (Sigma-Aldrich, MO, USA) and daptomycin (MedChemExpress, NJ, USA) were purchased from commercially available sources and prepared and stored according to manufacturer’s recommendations.

### LoC fabrication

Briefly, a 3D master mold for the cell culture chamber, measuring 1 × 20 × 0.25 mm (W × L × T), was designed using 3D modeling software (Autodesk Inventor) and fabricated with a 3D printer (Form 3B+; Formlabs, MA, USA), followed by surface treatment with trichloro(1H,1H,2H,2H-perfluorooctyl) silane (#448931; Sigma-Aldrich, MO, USA) for 40 min. A viscous pre-polymer, polydimethylsiloxane (PDMS; #4019862; Dow Silicones Corporation, United Kingdom), was prepared by mixing base polymer and curing agent at a 10:1 weight ratio, followed by degassing and casting directly into the 3D-printed mold affixed between two glass microscope slides (#3051; Clay Adams) for overnight curing at 60°C. After curing, the patterned PDMS layer was peeled off, aligned, and bonded to a microscope cover glass (#1169W12; Thomas Scientific LLC, PA, USA) following oxygen plasma treatment at 75 W and 100 millitorr oxygen (Tergeo Plasma Cleaner; PIE Scientific, CA, USA) for 60 seconds, which enabled the sealing of the cell culture chamber. To ensure strong covalent siloxane bonding between the PDMS and glass, the chips were then placed on a hot plate at 120°C for 30 min.

### LoC preparation for cell seeding

Prior to seeding, empty chips were washed with 70% ethanol followed by PBS before coating with either Matrigel (10 µg/mL) for 1 h at 37°C 5% CO_2_ for murine chips, or Matrigel (10 µg/mL) plus 0.1% gelatin solution (StemCell Technologies, Canada) for 1 h at 37°C 5% CO_2_ for human chips. After incubation with coating solution, sterilized PTFE tubing (Masterflex, Germany) was inserted into the channel inlets and outlets, followed by flushing with 100 µL ice-cold PBS. For murine cells, following live cell dye staining preparation as described above, each of the three cell types: LSECs, KCs, and HSCs were then diluted and combined to yield a suspension of 4 × 10^6^ LSECs/mL, 2 × 10^6^ KCs/mL, and 0.5 × 10^6^ HSCs/mL. For human LoCs, the same seeding densities were used; however, primary LSECs and HSCs were detached from culture flasks using 1X TrypLE Express (Thermo Fisher, MA, USA), and KCs were thawed upon use. The combined cell suspensions were then gently aspirated into a syringe with a 23-gage needle affixed, and slowly injected through the PTFE tubing to seed the chips. Final seeding density per chip was 2 × 10^4^ LSECs, 1 × 10^4^ KCs, and 0.5 × 10^3^ HSCs. Following confirmation of successful and uniform seeding across the channel of each chip, cells were allowed to adhere overnight at 37°C 5% CO_2_ in DMEM +10% FBS for murine chips and Complete Classic Medium for human chips. The next day, chips were connected to an Ismatec Reglo ICC Digital Pump (Masterflex, Germany) and introduced to flow conditions at 1 µL/min for 24 h prior to beginning experiments.

### LoC infection and treatment

Liquid cultures of *S. aureus* strain (GFP USA300 LAC) were prepared as previously described and diluted to 1.667 × 10^3^ CFU/mL for murine LoC and 8.335 × 10^3^ CFU/mL for human LoC in LoC culture media (24). Peristaltic pump tubing was then disconnected from the LoCs and media was cleared from the tubes. The tubing was then attached to syringes containing the bacterial inoculum, and 150 µL of the inoculum was drawn into the tubing, followed by the addition of 100 µL uninoculated media to allow for infection with roughly 250 CFU/chip for murine chips and 1250 CFU/chip for human chips. LoCs were then reconnected to the pump tubing, and infection was carried out at 2.5 µL/min for 1.5 h. Following infection, 100 µg/mL gentamicin-supplemented media was introduced to remove any remaining extracellular SA from the chips for 1 h at 2 µL/min. Media supplemented with either vancomycin (17 µg/mL) or daptomycin (37 µg/mL) simulating human exposure to achieve target attainment AUC:MIC ratios of 400 and 666, respectively, during SAB treatment were then introduced to the chips at 1 µL/min for 24 h for murine chips or 72 h for human chips (25–28). For metabolic control treatment experiments, murine LoCs were treated either with 2-deoxyglucose (2DG, 50 mM; Sigma-Aldrich, MA, USA) or a combination of oligomycin (10 µM), rotenone (5 µM), and antimycin A (1 µM) (ORAA; Agilent Technologies, CA, USA) for 1 h prior to fluorescence lifetime measurement.

### Cell viability assays

Cell viability in murine LoCs was carried out using a combination of Hoechst (5 µg/mL) (Thermo Fisher, MA, USA) and propidium iodide (25 µM) (Thermo Fisher, MA, USA) for 15 min and imaged on a Keyence BZ-X800 microscope, followed by analysis in Fiji.

### Live cell staining

Murine LSECs, HSCs, and KCs were stained just prior to seeding in liver chips by utilizing CellTracker CMTPX (Thermo Fisher, MA, USA), CellTracker CMTMR (Thermo Fisher, MA, USA), and BioTracker 655 (Sigma-Aldrich, MO, USA). Briefly, following cell lifting from culture flasks, cells were diluted to 6 × 10^6^ cells/mL for LSECs and HSCs, and 1 × 10^6^ cells/mL for KCs. Then, LSECs and HSCs were stained with CellTracker CMTPX (15 µg/mL) or CellTracker CMTMR (10 µg/mL) and incubated for 40 min at 37°C 5% CO_2_, and KCs stained with BioTracker 655 (5 µL/1 x 10^6^ cells) for 20 min at 37°C 5% CO_2_. After the incubation period, cells were washed by centrifugation at 300 × *g* for 5 min and PBS resuspension twice.

### Two-photon excited fluorescence lifetime imaging microscopy

Established murine liver chips were disconnected from flow before placing on the microscope stage of an SP8 two-photon laser scanning confocal microscope (Leica Microsystems) equipped with a pulsed (80 MHz, 100 fs pulse width) titanium–sapphire laser. TPE-FLIM images were acquired with a 63× objective lens for TPE-FLIM with excitation at 740 nm at 9.5% laser power, and subsequent detection at 450–490 nm. Time-correlated single photon counting was performed using an NDD port-coupled hybrid detector with an image size of 512 × 512 pixels, 10 frame repetitions per region of interest, and a pixel dwell time of 7.69 μs. To visualize CellTracker in the samples for cell type differentiation, TPE at 840 nm and emission detected from 560 to 680 nm in sequence immediately after NAD(*P*)H imaging with identical scan rate and repetition settings. After image acquisition, all images were processed in LASX software by pixel binning (pixel bin = 3) and intensity thresholding to 25 counts to remove background noise. Biexponential fitting was performed on fluorescence decay curves to determine intensity-weighted mean NAD(*P*)H lifetime (τm). Separately, phasor-guided masks were generated from the 840 nm TPE images and saved for later analysis of single-cell τm of liver cell subpopulations. Images containing τm data were then exported for further analysis in Fiji (29). CellPose3 with pretrained cyto3 model and deblur image restoration was used to generate masks from NAD(*P*)H intensity images and used in Fiji for segmenting τm data (30, 31). Any pixels within cell segments with 0 values (below the photon/pixel threshold count) were removed. Individual cell τm from each image for all treatment groups were then measured and recorded.

### Intracellular *S. aureus* survival assays

Determination of intracellular bacterial load following infection and antibiotic treatment was performed on a Keyence BZ-X800 fluorescence microscope (Fig. S5). Green fluorescent signal was measured using a GFP filter (470/40 nm excitation, 525/50 nm emission, OP-87763) at 20× magnification. Images were exported to Fiji, where fluorescence signal was processed through auto-thresholding using the RenyiEntropy method for determination of RFU (30, 32). Measurements were made at 24 h for murine LoCs, and 24 h, 48 h, and 72 h for human LoCs.

### Mitochondrial ROS assays

For human LoC experiments, mitochondrial ROS (mtROS) was measured at 72 h postinfection and treatment by fluorescence microscopy following live cell staining with mitoSOX red (Thermo Fisher, MA, USA) per manufacturer instructions. Briefly, chips were stained with 500 nM mitoSOX red for 30 min at 37°C 5% CO_2_ followed by gentle washing with 100 µL PBS using a pipette prior to imaging. mitoSOX red fluorescence was then measured on a Keyence BZ-X800 fluorescence microscope with a Texas Red filter (560/40 nm excitation, 630/75 nm emission, OP-87765). Images were exported and processed in Fiji as described above.

## RESULTS

### Establishing the liver-on-chip model

**Fig 1:**
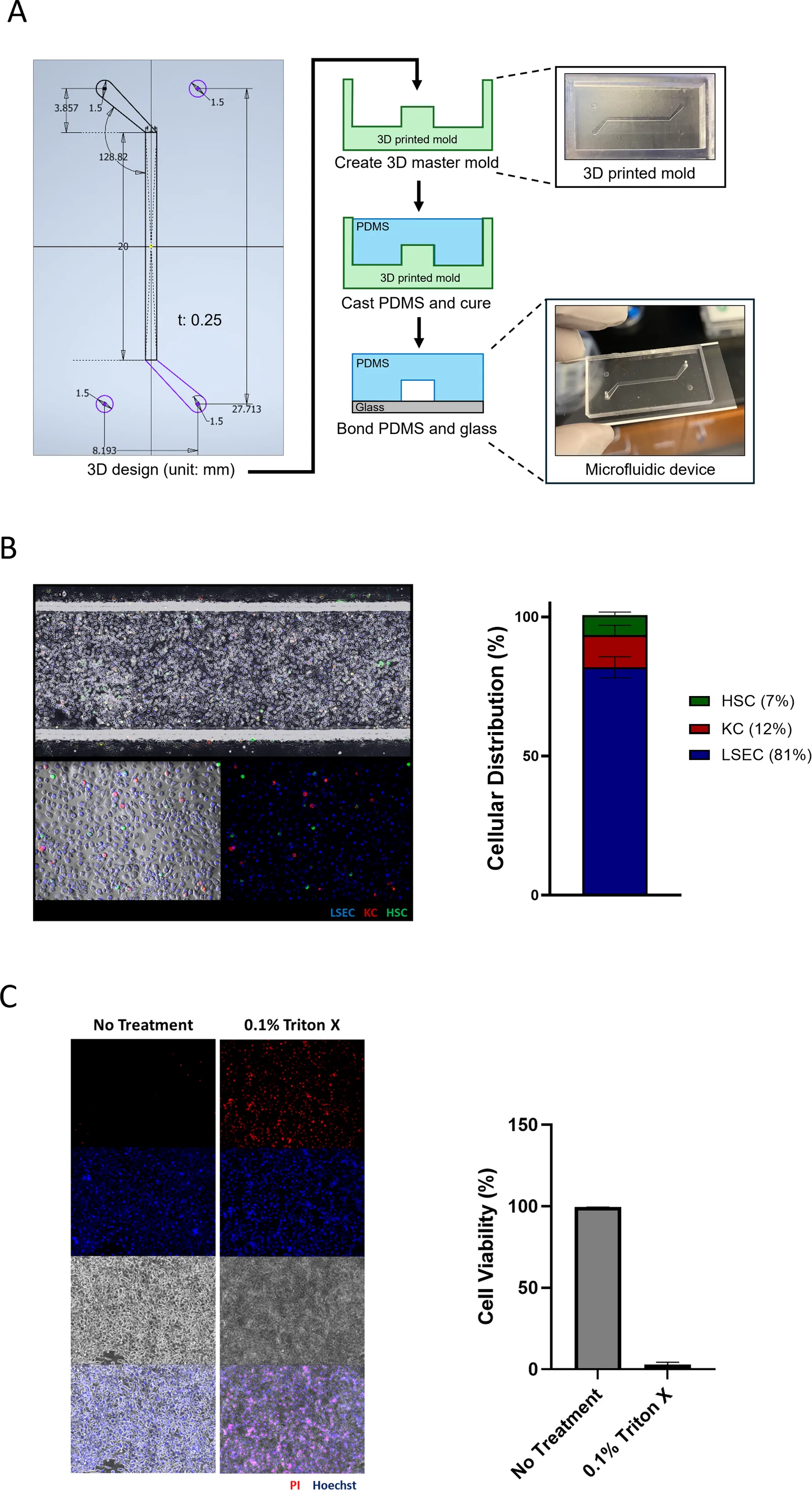
Chip fabrication and cellular spatial and viability characterization of murine liver-on-chip model. A 3D mold was generated for fabrication of PDMS chips bonded to microscope coverslip glass (**A**). Cells were stained with various fluorescent dyes (LSEC: Hoechst, KC: CellTracker CMTPX, HSC: CellTracker CMTMR) prior to seeding in liver chips. Following 2 days under flow conditions, cells were visualized *via* fluorescence microscopy to evaluate spatial distribution (**B**). Cell viability after the same time period was also determined utilizing a live/dead fluorescence staining assay with propidium iodide and Hoechst, and compared to a killing control with 0.1% Triton X (**C**). *n* = 3 ROIs for spatial distribution and *n* = 2 ROIs for viability determination.

A single-channel polydimethylsiloxane (PDMS)-based chip design was developed and fabricated using a 3D master mold, bonded to microscope cover glass slips (Fig. 1A). To establish a liver-on-chip model of SAB, pilot studies were conducted with murine LSEC, HSC, and KC cell lines to determine cellular distribution and cell viability in the chips following multiple days under mechanical flow conditions. A combination of Hoechst, CellTracker CMTMR, and CellTracker CMTPX was utilized to fluorescently differentiate LSECs, HSCs, and KCs, respectively, after 2 days under flow conditions (Fig. 1B). The combination of cells had become completely confluent within the microfluidic channels, and all three cell types were present throughout the channel. The cells remained adhered in the channel despite exposure to shear forces under flow conditions at 1 µL/min. Cell viability was determined to be 99.4% by live/dead fluorescence staining after 2 days under flow conditions (Fig. 1C). These positive readouts for retained cellular distribution and viability suggested cells could be sufficiently maintained for multiple days within the microphysiological system established on chip. Additionally, initial pilot studies demonstrated strong correlation between intracellular GFP RFU and intracellular CFU counts (Fig. S3), and that bacterial uptake was predominantly by KCs while a minority population of GFP-SA resided inside HSCs and LSECs (Fig. S4a and b).

### Two-photon-excited cellular differentiation of metabolic signals via FLIM

To determine the effects of infection and antibiotics on cellular metabolism within the liver-on-chip model, we aim to separate out metabolic signals by cell type. To achieve this, we sought to utilize fluorescent cell dyes to separately stain each of the three cell types in the liver-on-chip model to differentiate each cell for categorization of TPE-FLIM signals obtained subsequently. While the fluorescent dyes utilized do not have officially reported TPE parameters, in theory, fluorescent dyes can be excited using TPE. Thus, we empirically determined optimal TPE parameters to not only excite and capture emission of the three different cell tracking dyes, but to reliably separate the three different fluorescent dyes’ photon arrival times for accurate differentiation of cell types through phasor plotting, with photon arrival times clustering in three separate areas along the universal circle suggesting the presence of three monoexponential fluorescence decay populations (Fig. S1). With this empirical determination of TPE parameters to distinguish each of the three cell types, we could thus confidently characterize their individual contributions to TPE-FLIM metabolic signals in response to infection and antibiotic treatment in our liver-on-chip model.

### Small molecule metabolic inhibitors induce measurable changes in metabolism in the liver-on-chip model

**Fig 2:**
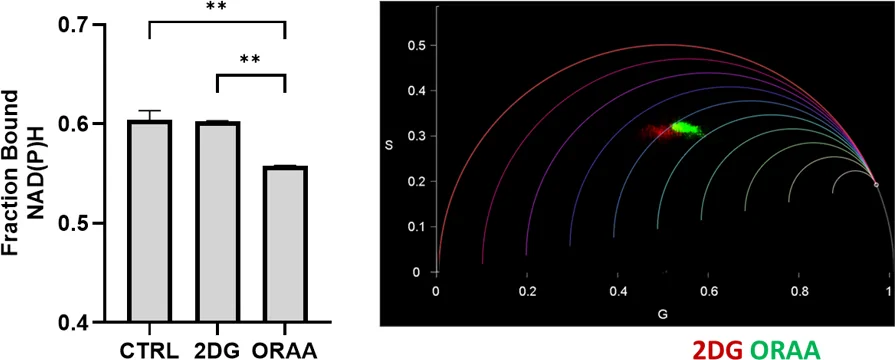
Metabolic control treatment effects on metabolic trajectory in murine liver-on-chip model as measured by TPE-FLIM. Murine liver chips were treated with either 2-deoxyglucose (2DG, 50 mM) or a combination of oligomycin (10 µM), rotenone (5 µM), and antimycin A (1 µM) (ORAA) for 1 h prior to fluorescence lifetime measurement. Fraction bound NAD(*P*)H was determined from phasor plots of photon arrival times between untreated, 2DG, and ORAA-treated murine liver chips (mean ± SD; *n* = 2). ** indicates *P < 0.01* in one-way ANOVA with Tukey’s *post hoc* analysis.

Using TPE-FLIM to measure changes in fluorescence lifetime of NAD(*P*)H provides information regarding cellular metabolic prioritization, namely, between preferential usage of glycolysis or oxidative phosphorylation. To determine whether changes in metabolism could be measured in our liver-on-chip model, we treated them with 2-deoxyglucose (2DG, a glucose analog that inhibits glycolysis) or a combination of oxidative phosphorylation inhibitors: oligomycin, rotenone, and antimycin A (inhibitors of ATP synthase, electron transport chain complex I, and complex III, respectively) (Fig. 2). We found that, compared to a no treatment control and 2DG treatment, the combination of OXPHOS inhibitors (ORAA) significantly decreased the measured fraction of bound NAD(*P*)H (0.56 vs 0.60, *P* = 0.0028, *P* = 0.0046, respectively), suggesting increased utilization of glycolysis when cellular OXPHOS was inhibited. The above findings confirmed that measurable changes in metabolic prioritization could be observed in our model. Further, fraction bound NAD(*P*)H within the tri-culture was significantly increased when cultured under flow *versus* static conditions, suggesting a more OXPHOS metabolic trajectory (Fig. S2).

### Evaluation of VAN and DAP on host metabolism and infection in murine liver-on-chip model

**Fig 3:**
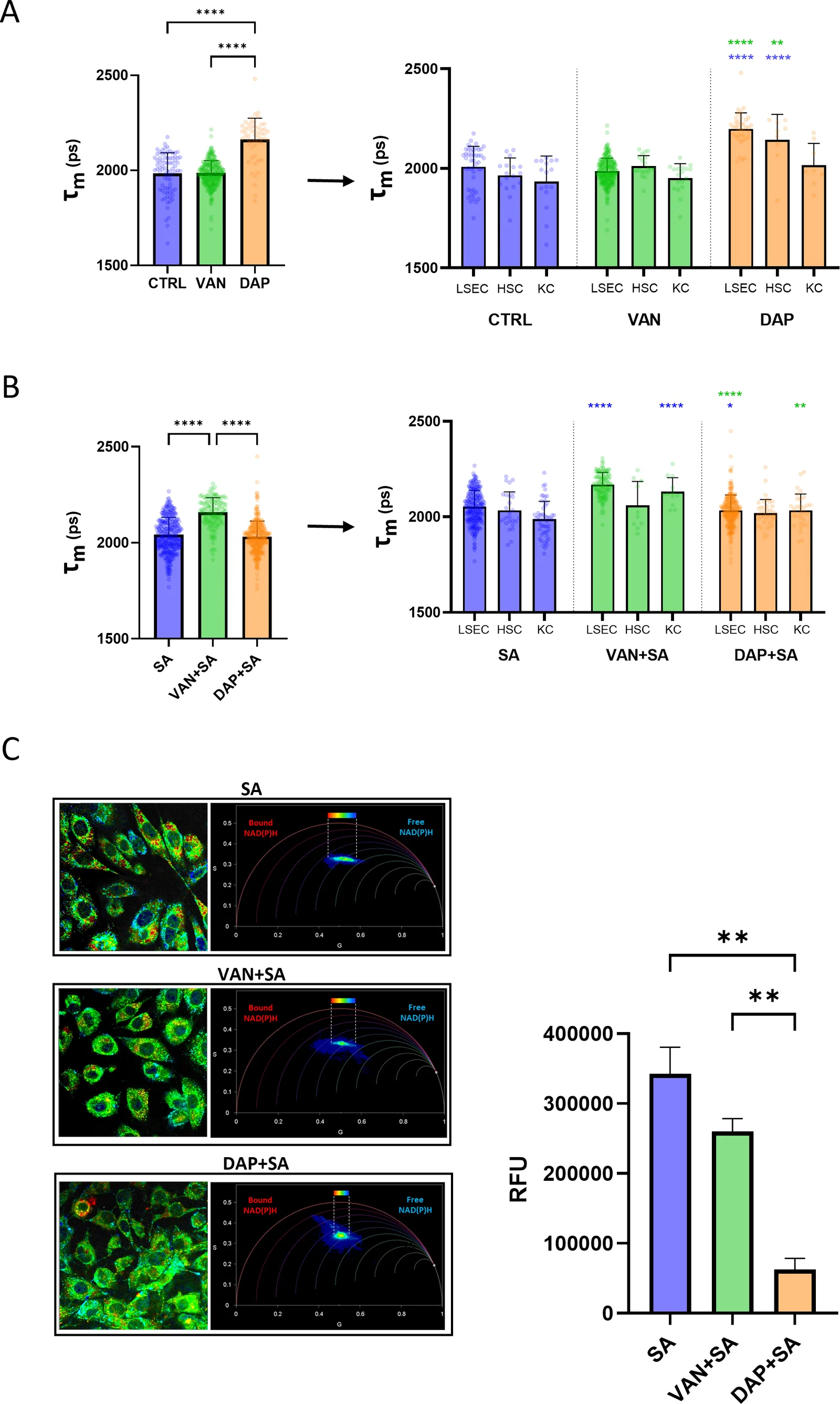
Immunometabolic effects and intracellular antimicrobial efficacy of vancomycin or daptomycin in the absence and presence of infection. Murine liver chips were treated with vancomycin (17 µg/mL) or daptomycin (37 µg/mL) for 24 h in the absence and presence of SA infection, followed by measurement of metabolic activity by TPE-FLIM. Single-cell mean NAD(*P*)H fluorescence lifetime values were determined in the absence of infection without antibiotic treatment, vancomycin, and daptomycin treatment, and mean NAD(*P*)H fluorescence lifetime was further differentiated by cell type based on co-localized fluorescence staining (**A**). Separately, single-cell mean NAD(*P*)H fluorescence lifetime values were determined in infected untreated, vancomycin, and daptomycin-treated liver chips (**B**). Photon arrival time was also visualized on phasor maps for each treatment for further visualization of population metabolic trajectory (**C**). Intracellular bacterial eradication efficacy was also compared among treatment groups in relation to infected-untreated control (SA) in murine liver chips *via* RFU determination of intracellular GFP (D). Images containing NAD(*P*)H fluorescence lifetime data were extracted to calculate mean single cell intensity-weighted lifetimes (τ_m_) for each treatment population [CTRL *n* = 80 (Liver sinusoidal endothelial cells, LSEC = 47; hepatic stellate cells, HSC = 17; Kupffer cells, KC = 16); VAN *n* = 281 (LSEC = 243; HSC = 20; KC = 18); DAP *n* = 55 (LSEC = 38; HSC = 9; KC = 8); SA *n* = 347 (LSEC = 268; HSC = 27; KC = 52); VAN+SA n =135 (LSEC = 113; HSC = 10; KC = 12); DAP+SA n =338 (LSEC = 267; HSC = 39; KC = 32)]. RFUs of intracellular aGFP SA were calculated (mean ± SD, *n* = 2 independent experiments). * indicates *P* < 0.05, ** indicates *P <* 0.01, and **** indicates *P <* 0.0001 in one-way ANOVA with Tukey’s *post hoc* analysis.

With the prior steps taken to characterize our liver-on-chip model, we then sought to determine how SA infection with or without antibiotic treatment (vancomycin or daptomycin) at physiologically relevant doses might impact host metabolic responses within the microphysiological liver sinusoidal environment. We used an epidemic MRSA strain (USA300 LAC) constitutively expressing GFP to enable spatiotemporal tracking of the infecting strain. Additionally, we sought to correlate metabolic responses with overall infection progression or resolution and compare to antibiotic treatment in the absence of infection in our model after 24 h. In the absence of infection, DAP treatment led to a significant increase in mean fluorescence lifetime (2163 ps vs 1984 ps, *P* < 0.0001) whereas VAN had no effect (1986 ps vs 1984 ps), suggesting a greater utilization of OXPHOS among the DAP-treated group (Fig. 3A). With further stratification of the single-cell metabolic FLIM data, the DAP group demonstrated a significant increase in mean fluorescence lifetime of LSECs (2198 ps vs 2008 ps, *P* < 0.0001) and HSCs (2143 ps vs 1964 ps, *P* < 0.0001), along with a slight but nonsignificant increase in lifetime among KCs (2016 ps vs 1933 ps, *P* = 0.1638) compared to untreated control.

After establishment of intracellular infection and subsequent antibiotic treatment under flow conditions for 24 h, we noted a statistically significant increase in overall mean fluorescence lifetime of the VAN-treated group compared to DAP-treated or untreated groups (2158 ps vs 2032 or 2042 ps, *P* < 0.0001), suggesting a greater utilization of OXPHOS among the vancomycin-treated group (Fig. 3B and C). Stratification of the single-cell metabolic FLIM data among infection groups exhibited a significant decrease in mean fluorescence lifetime of KCs with DAP treatment compared to VAN treatment (2034 ps vs 2132 ps, *P* = 0.0044) suggesting a trajectory toward glycolysis, as well as a significant decrease in LSEC mean fluorescence lifetime (2034 ps vs 2170 ps, *P* < 0.0001) and while HSC remained unchanged in mean fluorescence lifetime.

Infection progression or resolution was separately determined by fluorescence measurement of GFP fluorescence between treatment, where we observed a marked overall reduction in intracellular SA with DAP treatment compared to VAN treatment (62,395 RFU vs 260,148 RFU, *P* = 0.0095) corresponding to lower NAD(P)H fluorescence lifetime (Fig. 3D).

### Evaluation in human liver-on-chip model

**Fig 4:**
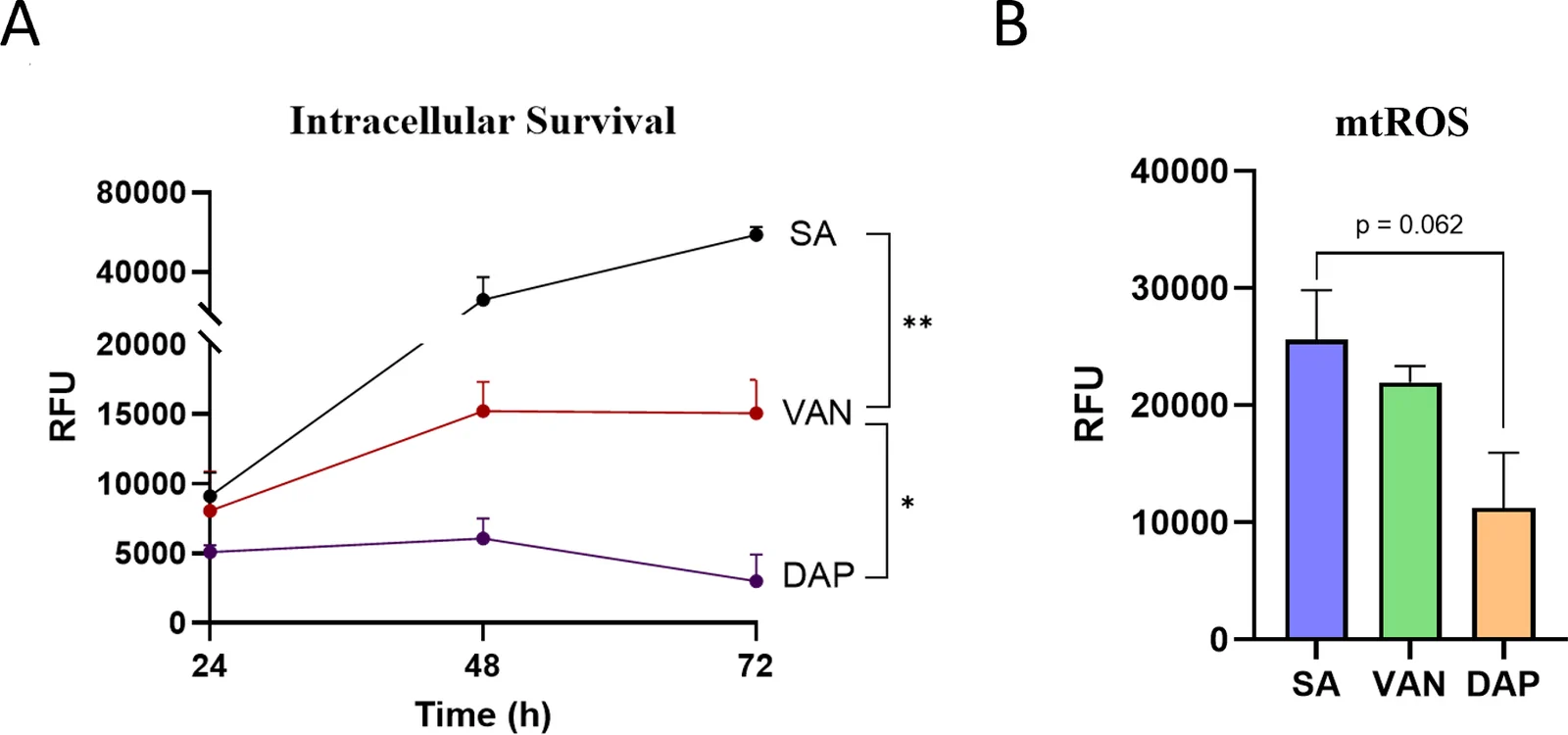
Effect of vancomycin or daptomycin on intracellular SA eradication and influence on host mitochondrial ROS (mtROS) production in a human liver-on-chip model. Liver chips seeded with primary human liver cells were infected and treated with vancomycin (17 µg/mL) or daptomycin (37 µg/mL) for 72 h under flow conditions to determine relative intracellular SA eradication (**A**) and effects on host mtROS production (**B**). Intracellular SA GFP RFUs and MitsoSOX Red RFUs were determined from 3 ROIs (mean ± SD, *n* = 2 independent experiments) to determine intracellular bacterial survival and mtROS production, respectively. * indicates *P* < 0.05 and ** indicates *P <* 0.01 in one-way ANOVA with Tukey’s *post hoc* analysis.

After establishing the LoC model using murine cells and observing the differences in metabolic trajectories corresponding to intracellular bacterial clearance between VAN and DAP, we evaluated infection dynamics and cellular immunometabolic response to antibiotic treatment on a human cell-based model. Here, we have extended infection out to 72 h as we have previously found that human primary KCs have a more robust antimicrobial response to SA infection, thereby minimizing the risk of bacterial overgrowth in our model. We tracked intracellular persistence of GFP-SA by fluorescence microscopy. As expected, we found that the overall burden of intracellular SA in our human chip model was quite low compared to the murine chip model, likely due to their comparatively more robust antimicrobial response to SA, and is suggestive of a low-level persistent infection which would likely be more representative of actual conditions within the human liver sinusoids during SAB. By 72 h, DAP-treated chips exhibited a significant reduction in intracellular SA compared to both VAN (3011 RFU vs 15,033 RFU, *P* = 0.0487) and untreated infected chips (3011 RFU vs 58,737 RFU, *P* = 0.0007) (Fig. 4A and Fig. S5). Furthermore, infection dynamics for DAP-treated chips exhibited a downward trend in presence of intracellular SA, with less SA at 72 h compared to 24 h, in contrast to VAN treatment which showed a slight increase in SA over the same period.

We also examined mitochondrial ROS (mtROS) production between treatment groups at 72 h to get a readout of the state of cellular immunometabolism under treatment relative to the observed differences in bacterial clearance (Fig. 4B). Of interest, a trend toward a reduction in mtROS associated with DAP treatment at day three compared to VAN (11,257 vs 21,945 RFU, *P =* 0.1252) and infected-untreated control (11,257 vs 25,592 RFU, *P* = 0.062) was observed, suggesting a redox-buffered state corresponding to infection resolution. Overall, these studies suggest treatment with DAP was better than VAN at achieving pathogen containment while preserving liver tissue metabolic homeostasis.

## DISCUSSION

Current clinical challenge with high morbidity and mortality of *S. aureus* bacteremia despite treatment with guideline-recommended antibiotics underscores the need to develop novel experimental models of infection that are more physiologically relevant in order to improve clinical translation. Organ-on-chip models are *in vitro* microphysiological systems that have made significant contributions in multiple disease areas in this regard. With the recent discovery of the pathogenic reservoir within the liver sinusoid and its critical contribution to the development of persistent infection, we sought to utilize this platform to better recapitulate this microenvironment in the pathogenesis of SAB to evaluate the efficacy of guideline-recommended antibiotics and the organ-level immunometabolic responses to treatment. We combined classic fluorescence methods for determination of intracellular SA clearance with novel methods (TPE-FLIM) to capture host immunometabolic response to infection and treatment. This approach allowed for a more comprehensive investigation of host-drug-microbe interactions that collectively contribute to infection dynamics within the liver sinusoid, and ultimately the outcome of the patient.

To our knowledge, we are the first to develop a liver-on-chip model of SA infection comprised of primary human cells derived exclusively from the liver sinusoid, capturing the multicellular complexity in the liver sinusoidal microenvironment to allow for in-depth organ-level evaluation of host immunometabolic responses to antibiotic treatment relative to intracellular SA eradication. Much of our initial efforts focused on characterizing this novel *in vitro* microphysiological model. While others have published work on SA infection utilizing a liver-on-chip model, the only liver cells used in the reported work were hepatocytes while the source of macrophages and endothelial cells were from blood monocytes and human umbilical vein, respectively (33). We demonstrated that all three liver cell types (LSECs, KCs, HSCs) maintained their viability and spatial distribution under flow conditions in our initial murine LoC model. We also showed that the cells within the LoC model produced expected measurable metabolic responses to inhibitors of glycolysis or OXPHOS, and that culture under flow conditions resulted in slight skewing toward OXPHOS metabolism compared to culture in static conditions. Cells grown *in vitro* typically exhibit greater reliance on glycolytic metabolism for ATP generation compared to cells within their native physiological environment, and the observed decrease in glycolytic prioritization associated with flow conditions in our model may demonstrate another feature that organ-on-chip models better recapitulate physiological conditions (34, 35). While the reliance on glycolytic energy production is generally considered intrinsic to immortalized cell lines, some prior evidence suggests that shear forces induce more reliance on OXPHOS in cell lines (36–38). Overall, our initial model characterization provided a foundation for the organ-level spatiotemporal tracking of SA and evaluation of antibiotic-mediated host immunometabolic responses and treatment efficacy against intracellular reservoirs of SA during bacteremia.

Our investigation of antibiotic-induced changes in host metabolic signature revealed differential effects of DAP and VAN in our infection model. Of interest, these effects were in contrast to those observed in our murine model in the absence of infection, where DAP in the absence of infection significantly increased NAD(*P*)H lifetime, while VAN had no effect. Different immunometabolic responses between uninfected and infected host cells are expected as immunometabolic changes may be induced by host-pathogen dynamics indirectly through altered microbial products, inflammation and host signaling or directly through off-target interactions with mammalian metabolic enzymes, mitochondria, or immunomodulatory pathways (3, 39, 40). Our initial experiments using murine LoC model demonstrated differences in antibiotic-mediated immunometabolic effects between uninfected and infected liver cells for both vancomycin and daptomycin. Since antibiotics are used for the treatment of infection, in our human-based model, we focused on the more relevant assessment of antibiotic treatment on immunometabolism of infected liver cells and explored further the production of mitochondrial ROS as a readout of cellular metabolic state relative to intracellular bacterial burden. We and others have previously described the connection between metabolic pathway prioritization and macrophage polarization; however, this complex culture system contains endothelial and mesenchymal cells which do not undergo polarization (2, 41). Yet, similarities do exist in their responses: LSECs have been shown to increase glycolytic prioritization in response to infection and contribute to the local inflammatory response (42). Further, they play an important role in coordinating immune cell recruitment and their subsequent responses during acute inflammation within the liver (43). Thus, we speculate that a more glycolytic metabolic state among LSECs in the presence of SA infection is beneficial as opposed to OXPHOS.

Utilization of live cell fluorescent dyes to differentiate cell types allowed us greater resolution into cellular immunometabolic responses to infection and antibiotic treatment. Notably, we are the first to take advantage of the use of TPE of cell-differentiating dyes in combination with NAD(*P*)H FLIM techniques to assess cell-type specific responses. Single-cell analysis indicated a significant decrease in LSEC NAD(*P*)H lifetime with DAP treatment, suggesting that daptomycin-mediated immunometabolic response of LSECs may contribute to the overall enhanced resolution of infection. However, with VAN treatment, a significant increase in NAD(*P*)H lifetime was observed, which may negatively impact the overall response to SA infection, especially in the *in vivo* environment where LSECs are relied on to effectively orchestrate antimicrobial responses of other circulating immune cells. A significant increase in KC NAD(*P*)H lifetime was also observed with VAN treatment, potentially suggesting a negative impact on KC pro-inflammatory polarization, while DAP-treated KCs were unaffected. When correlating these metabolic changes to relative intracellular SA clearance between DAP and VAN, the increased NAD(*P*)H lifetime observed with VAN treatment may be contributing to its overall lack of intracellular efficacy compared to DAP. Whereas DAP treatment retained the metabolic NAD(*P*)H FLIM signature of the infected-untreated group, while demonstrating significant efficacy in reducing intracellular SA burden. This contrasts with the metabolic effects of DAP on KCs we previously described in a KC monoculture co-infection model, where a significant increase in NAD(*P*)H fluorescence lifetime was observed (2). In the infected LoC model with flow, DAP did not significantly alter infected Kupffer cell NAD(*P*)H fluorescence lifetime, compared to the previously observed increase in the infected 2D culture model. This is more in line with what we would expect to see of the metabolic profile of macrophages engaging in bactericidal M1 polarized activities. In contrast, VAN in the LoC infection model, was associated with an increased NAD(*P*)H lifetime which would correspond to a metabolic trajectory that is less aligned with bactericidal function which in this case correlated with higher bacterial burden showing VAN to be inferior to DAP. However, it is notable that different timing, antibiotic concentrations, and inoculum were utilized in the respective experimental models. Inclusion of flow parameters in a LoC *versus* static conditions in the monoculture format may also contribute to the observed differences, since culture under flow was found to significantly alter metabolic trajectory in this study. Additionally, the inclusion of LSECs and HSCs in the LoC model allows for cell-cell interactions and angiocrine signaling that may influence KC metabolic responses to infection and antibiotic treatment. LSECs can contextually enhance or mitigate KC activation, and both LSECs and HSCs are critical in defining the “KC niche” (44, 45). Further, LSECs have been shown to increase chemokine and adhesion molecule production to recruit immune cells to the liver in a diphtheria toxin-mediated KC depletion model (46). The infected-untreated group is experiencing significant PAMP burden, likely driving strong inflammatory responses. Yet, this is insufficient to mount an effective immune response in the absence of antibiotics. The evidence here of DAP treatment retaining the metabolic profile while reducing PAMP load through direct intracellular antimicrobial efficacy, could be contributing to its overall superior efficacy against intracellular SA.

The inclusion of our human-based liver-on-chip model enhances the translatability of our findings. The starting inoculum was informed by reported median bacterial load in the blood of complicated SAB patients being 10^3^ CFU/mL (12). The overall bacterial burden with both these treatments in the human-based chip model is low, simulating the human bacteremic condition. Both our murine and human liver-on-chip models demonstrated the superiority of DAP over VAN in targeting the pathogenic reservoir in the liver sinusoid, with the human-based model extending infection out to 72 h to better recapitulate persistence in SAB. The progressive reduction in intracellular SA from 24 h to 72 h with DAP treatment is in clear contrast to the relatively sustained intracellular bacterial burden observed with VAN treatment. As the intracellular burden of SA decreases over time, the likelihood of SA dissemination from the pathogenic reservoir diminishes. In support of our findings, a recent meta-analysis of 20 clinical studies showed that early daptomycin use over vancomycin in patients with bacteremia caused by methicillin-resistant SA was associated with lower odds of mortality (47).

Production of mtROS can be indicative of cytotoxicity in certain settings, yet in the context of SAB, other investigators have observed production of mtROS as part of the innate immune response to SA, where it augments the antimicrobial action of the bacteria-containing phagolysosomes and contributes to the destruction of intracellular SA (48). As an endpoint readout in our model, however, increased mtROS in this functional context would perhaps be suggestive of a failure to clear the intracellular bacteria earlier during infection, and lower mtROS levels among the DAP treatment group might indicate a favorable immunometabolic profile suggestive of redox buffering in balancing pathogen containment with preservation of hepatic metabolic homeostasis.

While our liver-on-chip model and methods for evaluating antibiotic effects on both host and microbe offer new insights into treating SAB, there are several limitations to our approach. While this study offers a proof-of-concept for utilizing LoC models for the study of SAB, further validation of generated LoC models is important in determining specific contributions of this approach to *in vitro* modeling that improves our understanding of host-microbe responses to SAB. Future efforts to more fully characterize the implied cell-cell interactions with molecular and pathway-associated resolution such as that from single cell RNA sequencing would likely uncover novel drivers of disease outcomes. Additionally, efforts to evaluate even more physiologically relevant iterations of these models, such as through inclusion of shear stress at levels more closely recapitulating the *in vivo* sinusoidal environment, as well as inclusion of cellular organization, nutrient density, and oxygenation that correspond to those observed in hepatic zones (e.g., KCs predominantly localized to nutrient and oxygen-rich periportal zone 1), would also provide valuable insights for treating SAB. The flow rate of 1 µL/min utilized in our model provides shear stress of 0.016 dynes/cm^2^ to the endothelial sinusoidal compartment similar to those found in a commercially available LoC platform (Emulate Liver-Chip S1) that has recently been accepted into FDA’s ISTAND (Innovative Science and Technology Approaches for New Drugs) program. However, shear stress experienced by cells within the liver sinusoid are expected to be much greater, up to roughly four dynes/cm^2^ (49). Thus, our model faces the same limitation in this regard as others. Also, our model does not include all of the immune cells within the liver that contribute to the overall immune response. Future iterations that include circulating immune cells such as monocytes, neutrophils, and platelets could further enhance the model’s physiological relevance. Our finding that LSECs and HSCs are actively infected by SA deserves further investigation, as these cell types are not currently reported in the literature as major contributors to intracellular reservoirs of infection in SAB. Increasing the physiological relevance of this LoC model would allow for more comprehensive determination of the degree to which SA may be taken up by non-immune liver cells. While adhesion and invasion mechanisms such as fibronectin-integrin interactions are utilized by SA in microcapillary environments, their involvement within the liver may be minimized when investigated in a more physiologically relevant LoC model replete with a fully functional immune system (50, 51). Further, we acknowledge that utilization of murine cell lines to initially establish the model, and subsequent investigation of infection and antibiotic-induced metabolic effects *via* TPE-FLIM may not reflect metabolic changes observed in human primary cells. Nonetheless, similar intracellular efficacy for vancomycin and daptomycin was observed between our murine- and human cell-based models. A major benefit of TPE-FLIM is also in its label-free measurement of metabolism, one that lends itself to non-destructive kinetic measurements of metabolism. However, due to challenges with feasibility for establishing and maintaining the OoC platform and imaging at multiple time points, we were limited in obtaining dynamic insights into the temporal host immunometabolic response throughout the infection process. Also, while bacterial burden determination and metabolic imaging in this study were performed separately, future efforts to combine cell type stratification with bacterial proliferation dyes may uncover consequential effects of cell-specific intracellular SA reservoirs on auto and paracrine signaling during infection that could impact disease outcomes and treatment response. Finally, while TPE-FLIM is an innovative approach to measure NAD(*P*)H utilization as indirect readouts of metabolic response, inclusion of metabolomic analyses in parallel and molecular investigations to probe specific mechanisms in metabolic pathways would further enhance our understanding of how infection and antibiotic treatment impact host immunometabolism.

Our LoC model of SAB provides novel information on infection dynamics and targeting intracellular persistence, namely, the apparent effects of antibiotics on host metabolism during infection in the liver sinusoid that may contribute to their overall antibacterial efficacy during SAB. The observed superiority of DAP over VAN in both murine and human-based liver-on-chip models, as well as their differential impacts on host immunometabolic responses, provides further evidence that antibiotic selection in SAB would benefit from an updated approach that considers an antibiotic’s potential to clear intracellular SA within the liver sinusoid, as well as its potential host-directed effects which may further contribute to treatment efficacy. While clinical evidence has shown superiority of DAP over VAN, our findings provide additional information suggesting that DAP’s immunometabolic effects within the infected liver sinusoid may be contributing at least in part to better clinical outcomes in patients. Our approach to combine advanced microphysiological *in vitro* platforms with metabolic imaging provides a research platform to enhance our understanding of SAB, ultimately strengthening translatability of future findings to more effective treatment strategies in the clinic. Future antibiotic development may benefit from pre-clinical evaluation that incorporates antibiotic-mediated immunometabolic effects and intracellular efficacy in the disease microenvironment. As cellular metabolism governs immune cell function, a comprehensive assessment of the currently available antibiotics beyond their direct antimicrobial activity to include immunometabolic effects would reveal a more complete appreciation of the host-pathogen-drug interaction.

## Data Availability

The data sets used and/or analyzed during this study are available from the corresponding author upon reasonable request.

## SUPPLEMENTAL MATERIAL

Supplemental figures aac.00254-26-s0001.docx Fig. S1 to S5.

ASM does not own the copyrights to Supplemental Material that may be linked to, or accessed through, an article. The authors have granted ASM a non-exclusive, world-wide license to publish the Supplemental Material files. Please contact the corresponding author directly for reuse.

## References

[1] O’Neill LAJ, Kishton RJ, Rathmell J. 2016. A guide to immunometabolism for immunologists. Nat Rev Immunol 16:553–565. doi:10.1038/nri.2016.70 PMC5001910 27396447

[2] Beadell B, Wong-Beringer A. 2025. Effect of antibiotics on Kupffer cell immunometabolism relative to intracellular killing of *S. aureus* using NAD(P)H fluorescence lifetime imaging. mBio 16:e0212425. doi:10.1128/mbio.02124-25 PMC12607893 41025812

[3] Yang JH, Bhargava P, McCloskey D, Mao N, Palsson BO, Collins JJ. 2017. Antibiotic-induced changes to the host metabolic environment inhibit drug efficacy and alter immune function. Cell Host & Microbe 22:757–765. doi:10.1016/j.chom.2017.10.020 PMC5730482 29199098

[4] Krawczyk CM, Holowka T, Sun J, Blagih J, Amiel E, DeBerardinis RJ, Cross JR, Jung E, Thompson CB, Jones RG, Pearce EJ. 2010. Toll-like receptor-induced changes in glycolytic metabolism regulate dendritic cell activation. Blood 115:4742–4749. doi:10.1182/blood-2009-10-249540 PMC2890190 20351312

[5] Donnelly RP, Loftus RM, Keating SE, Liou KT, Biron CA, Gardiner CM, Finlay DK. 2014. mTORC1-dependent metabolic reprogramming is a prerequisite for NK cell effector function. J Immunol 193:4477–4484. doi:10.4049/jimmunol.1401558 PMC4201970 25261477

[6] Tannahill GM, Curtis AM, Adamik J, Palsson-McDermott EM, McGettrick AF, Goel G, Frezza C, Bernard NJ, Kelly B, Foley NH, et al.. 2013. Succinate is an inflammatory signal that induces IL-1β through HIF-1α. Nature 496:238–242. doi:10.1038/nature11986 PMC4031686 23535595

[7] Feaugas T, Sauvonnet N. 2021. Organ-on-chip to investigate host-pathogens interactions. Cell Microbiol 23:e13336. doi:10.1111/cmi.13336 33798273

[8] Lewis AJ, Seymour CW, Rosengart MR. 2016. Current murine models of sepsis. Surg Infect (Larchmt) 17:385–393. doi:10.1089/sur.2016.021 PMC4960474 27305321

[9] Wang N, Lu Y, Zheng J, Liu X. 2022. Of mice and men: laboratory murine models for recapitulating the immunosuppression of human sepsis. Front Immunol 13. doi:10.3389/fimmu.2022.956448 PMC9388785 35990662

[10] Stortz JA, Raymond SL, Mira JC, Moldawer LL, Mohr AM, Efron PA. 2017. Murine models of sepsis and trauma: can we bridge the gap? ILAR J 58:90–105. doi:10.1093/ilar/ilx007 PMC5886315 28444204

[11] Pollitt EJG, Szkuta PT, Burns N, Foster SJ. 2018. *Staphylococcus aureus* infection dynamics. PLoS Pathog 14:e1007112. doi:10.1371/journal.ppat.1007112 PMC6019756 29902272

[12] Nieman AE, Rozemeijer W, Savelkoul PHM, Schade RP. 2022. Bacterial DNA load in *Staphylococcus aureus* bacteremia is significantly higher in intravascular infections. Edited by A. Calderaro. PLoS One 17:e0266869. doi:10.1371/journal.pone.0266869 PMC9020692 35443013

[13] Turkeltaub L, Kashat L, Assous MV, Adler K, Bar-Meir M. 2024. Estimating bacterial load in *S. aureus* and *E. coli* bacteremia using bacterial growth graph from the continuous monitoring blood culture system. Eur J Clin Microbiol Infect Dis 43:1931–1938. doi:10.1007/s10096-024-04893-w PMC11405537 39073670

[14] Surewaard BGJ, Deniset JF, Zemp FJ, Amrein M, Otto M, Conly J, Omri A, Yates RM, Kubes P. 2016. Identification and treatment of the *Staphylococcus aureus* reservoir *in vivo*. J Exp Med 213:1141–1151. doi:10.1084/jem.20160334 PMC4925027 27325887

[15] Ortega-Prieto AM, Skelton JK, Wai SN, Large E, Lussignol M, Vizcay-Barrena G, Hughes D, Fleck RA, Thursz M, Catanese MT, Dorner M. 2018. 3D microfluidic liver cultures as a physiological preclinical tool for hepatitis B virus infection. Nat Commun 9:682. doi:10.1038/s41467-018-02969-8 PMC5813240 29445209

[16] Ahmed HMM, Salerno S, Morelli S, Giorno L, De Bartolo L. 2017. 3D liver membrane system by co-culturing human hepatocytes, sinusoidal endothelial and stellate cells. Biofabrication 9:025022. doi:10.1088/1758-5090/aa70c7 28548045

[17] Yang Y, Bai W, Dong J, Lv H, Zhu Y. 2022. Microflows in two-generation alveolar cells at an acinar bifurcation. Biomicrofluidics 16:054101. doi:10.1063/5.0098302 PMC9451617 36097514

[18] Alonso-Roman R, Mosig AS, Figge MT, Papenfort K, Eggeling C, Schacher FH, Hube B, Gresnigt MS. 2024. Organ-on-chip models for infectious disease research. Nat Microbiol 9:891–904. doi:10.1038/s41564-024-01645-6 38528150

[19] Ingber DE. 2020. Is it time for reviewer 3 to request human organ chip experiments instead of animal validation studies? Adv Sci (Weinh) 7:2002030. doi:10.1002/advs.202002030 PMC7675190 33240763

[20] Jang K-J, Otieno MA, Ronxhi J, Lim H-K, Ewart L, Kodella KR, Petropolis DB, Kulkarni G, Rubins JE, Conegliano D, et al.. 2019. Reproducing human and cross-species drug toxicities using a Liver-Chip. Sci Transl Med 11:eaax5516. doi:10.1126/scitranslmed.aax5516 31694927

[21] Ewart L, Apostolou A, Briggs SA, Carman CV, Chaff JT, Heng AR, Jadalannagari S, Janardhanan J, Jang K-J, Joshipura SR, et al.. 2022. Performance assessment and economic analysis of a human liver-chip for predictive toxicology. Commun Med 2:154. doi:10.1038/s43856-022-00209-1 PMC9727064 36473994

[22] Shi Q, Arefin A, Ren L, Papineau KS, Barnette DA, Schnackenberg LK, Hawes JJ, Avigan M, Mendrick DL, Ewart L, Ronxhi J. 2022. Co-culture of human primary hepatocytes and nonparenchymal liver cells in the emulate liver-chip for the study of drug-induced liver injury. Curr Protoc 2:e478. doi:10.1002/cpz1.478 35790095

[23] Kitani H, Sakuma C, Takenouchi T, Sato M, Yoshioka M, Yamanaka N. 2014. Establishment of c-myc-immortalized kupffer cell line from a C57BL/6 mouse strain. Results Immunol 4:68–74. doi:10.1016/j.rinim.2014.08.001 PMC4213843 25379377

[24] Beadell B, Yamauchi J, Wong-Beringer A. 2024. Comparative *in vitro* efficacy of antibiotics against the intracellular reservoir of *Staphylococcus aureus*. J Antimicrob Chemother 79:2471–2478. doi:10.1093/jac/dkae241 PMC11441993 39073778

[25] Holmes NE, Turnidge JD, Munckhof WJ, Robinson JO, Korman TM, O’Sullivan MVN, Anderson TL, Roberts SA, Warren SJC, Gao W, Howden BP, Johnson PDR. 2013. Vancomycin AUC/MIC ratio and 30-day mortality in patients with *Staphylococcus aureus* bacteremia. Antimicrob Agents Chemother 57:1654–1663. doi:10.1128/AAC.01485-12 PMC3623342 23335735

[26] Urakami T, Hamada Y, Oka Y, Okinaka T, Yamakuchi H, Magarifuchi H, Aoki Y. 2019. Clinical pharmacokinetic and pharmacodynamic analysis of daptomycin and the necessity of high-dose regimen in Japanese adult patients. J Infect Chemother 25:437–443. doi:10.1016/j.jiac.2019.01.011 30782427

[27] Falcone M, Russo A, Cassetta MI, Lappa A, Tritapepe L, d’Ettorre G, Fallani S, Novelli A, Venditti M. 2013. Variability of pharmacokinetic parameters in patients receiving different dosages of daptomycin: is therapeutic drug monitoring necessary? J Infect Chemother 19:732–739. doi:10.1007/s10156-013-0559-z 23361566

[28] Olney KB, Howard JI, Burgess DS. 2024. Daptomycin dose optimization in pediatric *Staphylococcus aureus* bacteremia: a pharmacokinetic/pharmacodynamic investigation. J Clin Pharmacol 64:860–865. doi:10.1002/jcph.2425 38497326

[29] Schindelin J, Arganda-Carreras I, Frise E, Kaynig V, Longair M, Pietzsch T, Preibisch S, Rueden C, Saalfeld S, Schmid B, Tinevez J-Y, White DJ, Hartenstein V, Eliceiri K, Tomancak P, Cardona A. 2012. Fiji: an open-source platform for biological-image analysis. Nat Methods 9:676–682. doi:10.1038/nmeth.2019 PMC3855844 22743772

[30] Stringer C, Wang T, Michaelos M, Pachitariu M. 2021. Cellpose: a generalist algorithm for cellular segmentation. Nat Methods 18:100–106. doi:10.1038/s41592-020-01018-x 33318659

[31] Stringer C, Pachitariu M. 2025. Cellpose3: one-click image restoration for improved cellular segmentation. Nat Methods 22:592–599. doi:10.1038/s41592-025-02595-5 PMC11903308 39939718

[32] Kapur JN, Sahoo PK, Wong AKC. 1985. A new method for gray-level picture thresholding using the entropy of the histogram. Computer Vision, Graphics, and Image Processing 29:273–285. doi:10.1016/0734-189X(85)90125-2

[33] Siwczak F, Cseresnyes Z, Hassan MIA, Aina KO, Carlstedt S, Sigmund A, Groger M, Surewaard BGJ, Werz O, Figge MT, Tuchscherr L, Loffler B, Mosig AS. 2022. Human macrophage polarization determines bacterial persistence of *Staphylococcus aureus* in a liver-on-chip-based infection model. Biomaterials 287:121632. doi:10.1016/j.biomaterials.2022.121632 35728409

[34] Thompson CL, Fu S, Knight MM, Thorpe SD. 2020. Mechanical stimulation: a crucial element of organ-on-chip models. Front Bioeng Biotechnol 8:602646. doi:10.3389/fbioe.2020.602646 PMC7758201 33363131

[35] Chen P-H, Cai L, Huffman K, Yang C, Kim J, Faubert B, Boroughs L, Ko B, Sudderth J, McMillan EA, et al.. 2019. Metabolic diversity in human non-small cell lung cancer cells. Mol Cell 76:838–851. doi:10.1016/j.molcel.2019.08.028 PMC6898782 31564558

[36] Rehberg M, Ritter JB, Reichl U. 2014. Glycolysis is governed by growth regime and simple enzyme regulation in adherent MDCK cells. PLoS Comput Biol 10:e1003885. doi:10.1371/journal.pcbi.1003885 PMC4211564 25329309

[37] Simões-Faria R, Daems M, Peacock HM, Declercq M, Willems A, Jones EAV, Ghesquière B. 2025. Wall shear stress modulates metabolic pathways in endothelial cells. Metabolomics 21:16. doi:10.1007/s11306-024-02214-y PMC11753319 39832080

[38] Chiu JJ, Chien S. 2011. Effects of disturbed flow on vascular endothelium: pathophysiological basis and clinical perspectives. Physiol Rev 91:327–387. doi:10.1152/physrev.00047.2009 PMC3844671 21248169

[39] Kanoh S, Rubin BK. 2010. Mechanisms of action and clinical application of macrolides as immunomodulatory medications. Clin Microbiol Rev 23:590–615. doi:10.1128/CMR.00078-09 PMC2901655 20610825

[40] Moullan N, Mouchiroud L, Wang X, Ryu D, Williams EG, Mottis A, Jovaisaite V, Frochaux MV, Quiros PM, Deplancke B, Houtkooper RH, Auwerx J. 2015. Tetracyclines disturb mitochondrial function across eukaryotic models: a call for caution in biomedical research. Cell Rep 10:1681–1691. doi:10.1016/j.celrep.2015.02.034 PMC4565776 25772356

[41] Marrocco A, Ortiz LA. 2022. Role of metabolic reprogramming in pro-inflammatory cytokine secretion from LPS or silica-activated macrophages. Front Immunol 13:936167. doi:10.3389/fimmu.2022.936167 PMC9633986 36341426

[42] Aparicio-Fernandez L, Antoran A, Areitio M, Rodriguez-Erenaga O, Martin-Souto L, Buldain I, Márquez J, Benedicto A, Arteta B, Pellon A, Moyes DL, Rementeria A, Ramirez-Garcia A. 2024. Candida albicans increases the aerobic glycolysis and activates MAPK-dependent inflammatory response of liver sinusoidal endothelial cells. Microbes Infect 26:105305. doi:10.1016/j.micinf.2024.105305 38296157

[43] Papaioannou S, See J-X, Jeong M, De La Torre C, Ast V, Reiners-Koch P-S, Sati A, Mogler C, Platten M, Cerwenka A, Stojanovic A. 2023. Liver sinusoidal endothelial cells orchestrate NK cell recruitment and activation in acute inflammatory liver injury. Cell Rep 42:112836. doi:10.1016/j.celrep.2023.112836 37471222

[44] Gao J, Lan T, Kostallari E, Guo Y, Lai E, Guillot A, Ding B, Tacke F, Tang C, Shah VH. 2024. Angiocrine signaling in sinusoidal homeostasis and liver diseases. J Hepatol 81:543–561. doi:10.1016/j.jhep.2024.05.014 PMC11906189 38763358

[45] Bonnardel J, T’Jonck W, Gaublomme D, Browaeys R, Scott CL, Martens L, Vanneste B, De Prijck S, Nedospasov SA, Kremer A, Van Hamme E, Borghgraef P, Toussaint W, De Bleser P, Mannaerts I, Beschin A, van Grunsven LA, Lambrecht BN, Taghon T, Lippens S, Elewaut D, Saeys Y, Guilliams M. 2019. Stellate cells, hepatocytes, and endothelial cells imprint the kupffer cell identity on monocytes colonizing the liver macrophage niche. Immunity 51:638–654. doi:10.1016/j.immuni.2019.08.017 PMC6876284 31561945

[46] Sakai M, Troutman TD, Seidman JS, Ouyang Z, Spann NJ, Abe Y, Ego KM, Bruni CM, Deng Z, Schlachetzki JCM, Nott A, Bennett H, Chang J, Vu BT, Pasillas MP, Link VM, Texari L, Heinz S, Thompson BM, McDonald JG, Geissmann F, Glass CK. 2019. Liver-derived signals sequentially reprogram myeloid enhancers to initiate and maintain kupffer cell identity. Immunity 51:655–670. doi:10.1016/j.immuni.2019.09.002 PMC6800814 31587991

[47] Adamu Y, Puig-Asensio M, Dabo B, Schweizer ML. 2024. Comparative effectiveness of daptomycin versus vancomycin among patients with methicillin-resistant *Staphylococcus aureus* (MRSA) bloodstream infections: a systematic literature review and meta-analysis. PLoS One 19:e0293423. doi:10.1371/journal.pone.0293423 PMC10881006 38381737

[48] Cohen TS, Boland ML, Boland BB, Takahashi V, Tovchigrechko A, Lee Y, Wilde AD, Mazaitis MJ, Jones-Nelson O, Tkaczyk C, Raja R, Stover CK, Sellman BR. 2018. *S. aureus* evades macrophage killing through NLRP3-dependent effects on mitochondrial trafficking. Cell Rep 22:2431–2441. doi:10.1016/j.celrep.2018.02.027 PMC7160668 29490278

[49] Wang T, Lü S, Hao Y, Su Z, Long M, Cui Y. 2021. Influence of microflow on hepatic sinusoid blood flow and red blood cell deformation. Biophys J 120:4859–4873. doi:10.1016/j.bpj.2021.09.020 PMC8595567 34536388

[50] Schröder A, Schröder B, Roppenser B, Linder S, Sinha B, Fässler R, Aepfelbacher M. 2006. *Staphylococcus aureus* fibronectin binding protein-A induces motile attachment sites and complex actin remodeling in living endothelial cells. Mol Biol Cell 17:5198–5210. doi:10.1091/mbc.e06-05-0463 PMC1679684 17021255

[51] Laschke MW, Kerdudou S, Herrmann M, Menger MD. 2005. Intravital fluorescence microscopy: a novel tool for the study of the interaction of *Staphylococcus aureus* with the microvascular endothelium *in vivo*. J Infect Dis 191:435–443. doi:10.1086/427193 15633103

