# Supplementary material for: Using a liver-on-chip model to evaluate the effects of vancomycin and daptomycin on host immunometabolism and clearance of intracellular reservoirs of Staphylococcus aureus during bacteremia
Source: Antimicrob Agents Chemother. 2026 Jul 23;70(9):e00254-26. doi: 10.1128/aac.00254-26 (PMC13712974; doi:10.1128/aac.00254-26)
Supplement: Supplementary file 1 [file aac.00254-26-s0001.docx]

**Figure S1**

**Figure S1.** Two photon excitation-fluorescence lifetime imaging microscopy (TPE-FLIM) excitation and photon arrival phasor plot of live-cell fluorescent dyes for cell differentiation. Liver sinusoidal endothelial cells (LSECs), hepatic stellate cells (HSCs), and Kupffer cells (KCs) were stained with CellTracker CMTPX (15 µg/mL), CellTracker CMTMR (10 µg/mL), and Biotracker 655 (5 µL/1 x 10^6^ cells), respectively, followed by two-photon excitation to determine spatial separation of photon arrival times *via* phasor plot (**a**). These were compared to monocultures for TPE comparison (**b**).

**Figure S2**

**Figure S2.** Effects of flow conditions on metabolic activity of cells in murine liver chips. Murine liver chips were seeded and metabolic activity was characterized *via* two photon excitation – fluorescence lifetime imaging microscopy (TPE-FLIM) in the presence and absence of flow conditions. Fraction bound NAD(P)H was determined in flow (0.016 dynes/cm^2^) and static conditions to determine effects on metabolic trajectory of murine cells within liver chips (mean ± SD; n = 2 independent experiments). **** indicates *p <* 0.01 in two-tailed Student’s *t* test.

**Figure S3**

**Figure S3*.*** Correlation between intracellular GFP SA RFUs and CFU enumeration. After establishing intracellular infection with GFP SA, RFUs were quantified followed by cell lysis and plating on TSA plates for bacterial enumeration to determine correlation between intracellular RFUs and CFUs. Shown are mean ± S.D. (n = 3).

**Figure S4**

**Figure S4*.*** *Intracellular SA uptake in LoC.* Images shown are of HSCs from the human LoC model with intracellular SA present (a), and SA uptake by cell type in the murine LoC model (b) (n = 142 total cells).

**Figure S5**

**Figure S5*.*** *Representative human liver-on-chip microscopy images illustrating intracellular S. aureus* (SA) infection. Images shown are of established human primary liver cells: liver sinusoidal endothelial cells (LSECs), hepatic stellate cells (HSCs), and Kupffer cells (KCs) on chip prior to infection were taken at 10X magnification (a), 48 h after initial infection at 20X magnification (b), and region of interests (ROIs) from 20X magnification images illustrating intracellular presence of *gfp* SA (c-f).
